# A conversation with Daniel M. Croymans, MD, MBA, MS, Medical Director of Quality, Department of Medicine, University of California Los Angeles

**DOI:** 10.1017/cts.2022.483

**Published:** 2022-12-27

**Authors:** Kathy Siranosian

**Affiliations:** Clinical Research Forum, Washington, DC, USA

## Top 10 Clinical Research Achievement Awards Q & A

This article is part of a series of interviews with recipients of Clinical Research Forum’s Top 10 Clinical Research Achievement Awards. This article is with Daniel M. Croymans, MD, MBA, MS, Medical Director of Quality, Department of Medicine, University of California Los Angeles. Dr. Croymans and his co-investigators tested the effect of behavioral interventions on the uptake of COVID-19 vaccines. Dr. Croymans received a 2022 Top 10 Clinical Research Achievement Award for *Behavioural nudges increase COVID-19 vaccinations* [1]. *The interview has been edited for length and clarity.*

## How Did You First Get Involved with Clinical Research?

Looking back, it’s clear that the end of my undergraduate training was a pivotal time for me. After finishing my degree in physiological science at UCLA, I planned to do volunteer work in Peru for a few months. Instead, one of my former professors reached out and asked if I could help move his lab from USC, where he was doing research, to UCLA, where he was teaching. It was an exercise and metabolic disease research lab, and I spent the entire winter break working on the move – 12, 15 h a day. The experience turned out to be like a crash course on how to set up a lab and run clinical trials combined with incredible one-on-one mentorship. It got me really excited about the field, and I ended up working on a trial about using resistance training as a means to improve markers of inflammation, future cardiovascular disease risk, and diabetes in obese, sedentary individuals. After that, I stayed at UCLA to get my masters in integrative physiology and biology.

## And Then You Went on to Earn an M.D. and an M.B.A.?

Yes, while working on my masters I realized that as much as I liked finding clinical research insights, what I enjoyed most was the implementation side, being able to help people change behaviors. We know that 70% of all health care utilization is driven by chronic disease management, and we also know that lifestyle choices drive a lot of chronic diseases. As I started to think more deeply about what impacts human choice, I became interested in behavioral science and that led me to medical school. Then, when I was in medical school, I realized that if I wanted to impact human choice at scale, I needed to better understand systems – and more specifically, the economics and finance of systems. That’s what drove me to get an MBA. I wanted to learn how we could apply our insights from behavioral science to drive effective and healthy decision-making at scale.

## It’s Almost Like You were Continually Discovering Different Pieces of a Puzzle and Figuring Out How They Fit Together?

Yes, but when I started thinking about how to apply behavioral science insights to healthy decision-making at scale, I realized that there was one piece missing: technology. Technology is key. That’s when I started to explore informatics and which technologies and systems could be used to impact human choice at scale.

## Is That What Led to Your Research on How Behavioral Nudges Impact COVID-19 Vaccinations?

Ultimately, yes. All four of my degrees are from UCLA, and I also did all of my training and got my first job out of residency there. So when the pandemic struck, I was UCLA’s Medical Director of Quality and had already started to bring together experts from a variety of different disciplines to have conversations about how we could apply behavioral science insights at scale in the healthcare environment. Once the COVID vaccine was developed, the biggest problem we needed to solve was how to encourage our UCLA patients to get vaccinated. We had a phenomenal team build out the logic around identifying eligibility from electronic health record (EHR) data, and then, we started to think about this as an opportunity to identify the best way to communicate the value of a vaccine in a way that drives people to obtain one. That became our research project, and we were able to show that text-based reminders can effectively encourage vaccinations across different demographic groups, with effects persisting for at least 8 weeks.

## Where is This Research Headed Next?

The area that we’re focusing on now is a communication program we call “My Action Plan” or MAP. Essentially, what we’re designing is a personalized action plan for every primary care patient at UCLA. It uses a patient’s EHR to identify care gaps and then sends a text message that is both informative and actionable for closing those gaps. MAP is taking what we learned from our research with COVID vaccines and expanding that to other key care gaps. What we’re learning is that if we want to get people in for their check-ups and screenings, we need to do that in a way that’s personable and actionable – so it’s not just, “you’re overdue for a mammogram,” but also “This is how to schedule your appointment.”

## So Once Again, You’re Designing Ways to Fit Together Those Puzzle Pieces – Behavioral Science, Patient Care, Health Systems, and Technology?

Those four factors are becoming increasingly intertwined in the healthcare environment. Retailers and other initiatives have been using mobile device technologies to capture user attention and drive awareness for years. It’s time for health systems to do the same, to know people’s preferences and deliver information that is both meaningful and actionable. Health systems are still far behind in this area, but this is the direction we need to go. Messages have to come to patients from the right person, at the right time, in a way that drives action.

## What’s Needed for Health Systems to Develop Better Communication Strategies for Promoting Health-Related Behaviors?

Investment in these types of solutions, for sure. But that’s not all. There also needs to be effective interdisciplinary collaboration. The challenges of developing better communication strategies are just too complex to be solved by a single expert in any one given area. The answer isn’t pure math, or pure physics, or any one thing. So, if you want to do this work and really make a difference, you need teamwork. The paper that won the award and our subsequent papers are emblematic of that. Our research involves PhD students and faculty in behavioral science, statisticians, health service researchers, clinicians, operators, and programmers to help us with the logic, others who understood the mobile interface – and all of us have to work together on the same page. I’m lucky in that I speak enough of everybody’s language to bring them all together, recognize their different motivations, and help everyone keep working toward our shared goal. There are four critical motivations that drive human beings – mastery (wanting to demonstrate excellence), relatedness (being part of a group working toward a common goal), purpose (that feeling that your work has meaning), and autonomy. Team leaders need to understand those core motivations and make sure that they’re being addressed. It can be hard work bringing great people together to do great things but going forward, that’s what it’s going to take. Collaboration is the foundation for effective interventions to improve patient care and health.
